# Dissociating the effects of alternative early-life feeding schedules on the development of adult depression-like phenotypes

**DOI:** 10.1038/s41598-017-13776-4

**Published:** 2017-11-01

**Authors:** Vikki Neville, Clare Andrews, Daniel Nettle, Melissa Bateson

**Affiliations:** 1 Institute of Neuroscience and Centre for Behaviour and Evolution, Newcastle University, Henry Wellcome Building, Framlington Place, Newcastle upon Tyne, NE2 4HH UK; 2 Present Address: School of Veterinary Science, University of Bristol, Bristol, UK

## Abstract

Early-life adversity is associated with increased vulnerability to depression in humans, and depression-like phenotypes in animals. However, different types of adverse experience may leave different signatures in adulthood. We experimentally manipulated the Amount of food delivered to European starling nestlings and the begging Effort required to obtain food during early development. Here, we report behavioural data in adulthood from a task that assessed sensitivity to shifts in reward magnitude characteristic of depression-like low mood. Birds that had experienced Hard Effort were more food motivated than birds that had experienced Easy Effort. Both Effort and Amount affected sensitivity to shifts in reward magnitude: Hard Effort birds showed an enhanced negative contrast effect following loss of reward (‘disappointment’), and Lean Amount birds failed to show a normal positive contrast effect following gain in reward (a lack of ‘elation’). Therefore, the feeding schedule experienced for just 10 days in early life caused enduring effects on feeding motivation and sensitivity to reward loss/gain consistent with human depression. Furthermore, the contrast effects were specific to different types of adversity. These results highlight the importance of early-life feeding schedules in the development of depression-like phenotypes.

## Introduction

**Figure 1:**
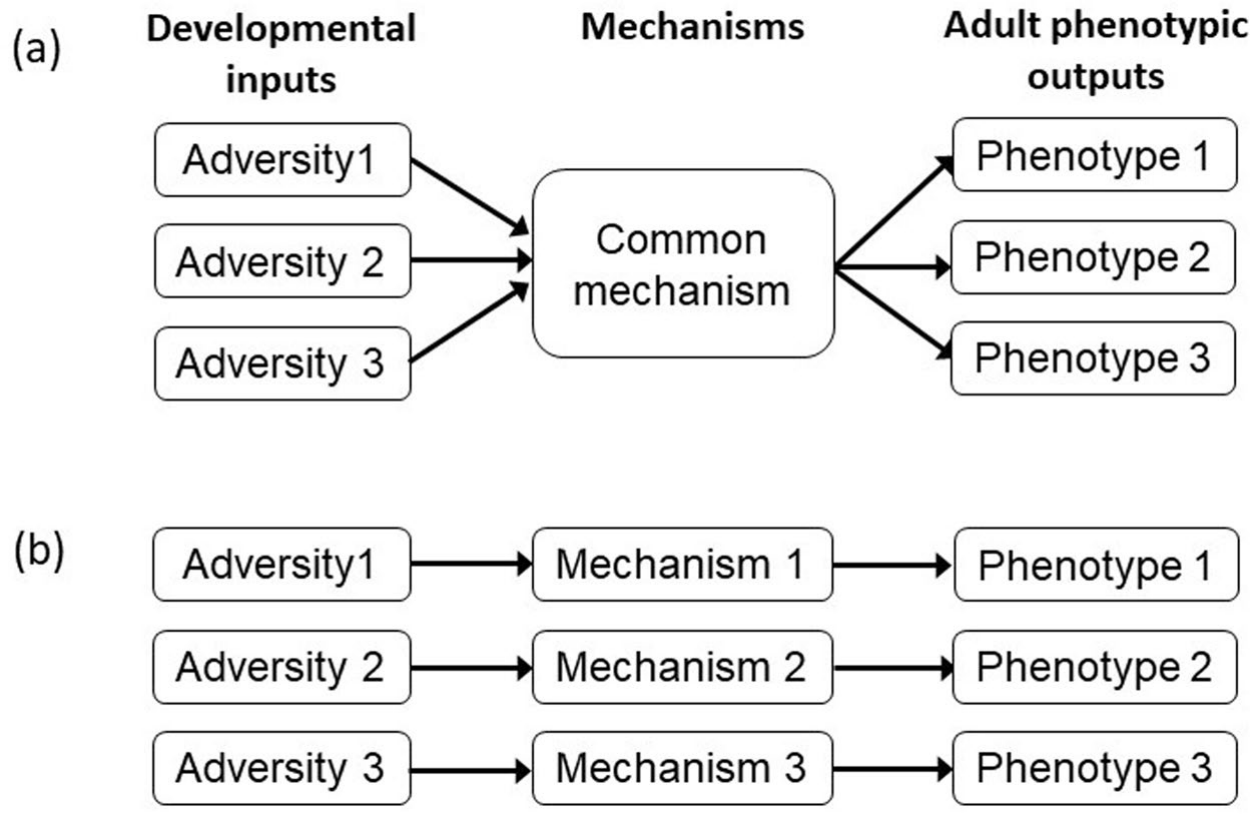
Two models of development. (**a**) The ‘common pathway’ model, whereby adult phenotypic traits are the output of a common developmental mechanism, which can be triggered by a number of different sources of developmental adversity. (**b**) The ‘specific effects’ model, whereby adult phenotypic traits are the output of different mechanisms, each caused by specific sources of developmental adversity.

In humans, various types of early-life adversity including physical and emotional neglect, abuse and trauma are associated with increased vulnerability to mood disorders in later life^1–3^. We propose two possible models of the association between early-life adversity and an adult depressive phenotype. According to a ‘common pathway’ model (Fig. 1a), different types of adversity all feed into a common mechanism that in turn influences the adult phenotype in a range of different ways (e.g. calibration of the developing HPA axis or disruption of hippocampal structure^4,5^). Alternatively, according to a ‘specific effects’ model (Fig. 1b), specific types of early-life adversity cause specific phenotypic outcomes via a range of different mechanisms^6,7^. Recent evidence hints that the type and timing of childhood exposure to adversity could be critical in determining its subsequent consequences for the development of mood disorders^8^. However, due to the fact that different types of adversity tend to be correlated in humans^6,9^, it is challenging to conclusively identify specific effects using epidemiological human data. Animal studies in which different sources of adversity are experimentally manipulated are necessary to conclusively test causal hypotheses.

Substantial effort has been devoted to the creation and study of experimental animal models of the effects of early-life adversity in laboratory rodents^10,11^. Many of these models involve manipulations in which pups are temporarily separated from the mother for varying periods in the first two weeks after birth^10^. In the light of the discussion above, one criticism of these rodent paradigms is that, like the human epidemiological studies, they typically confound different sources of adversity (e.g. malnutrition, hypothermia, lack of grooming, physical contact and protection). Few rodent studies have attempted to experimentally dissociate the effects of different developmental adversities on subsequent phenotypes. In one exception, Crnic *et al*.^12^ tested the moderating effects of providing rat pups removed from their mother and deprived of food for 12 hours a day with a non-lactating aunt. While some differences in behaviour and in brain biochemistry were found between the isolated pups and those placed with an aunt, the greatest differences were between both of the food-deprived groups and control pups left with the mother. This study can be interpreted as suggesting that food deprivation has a greater effect than social isolation alone. Studies of rats and mice in which dams are deprived of food during pregnancy and/or lactation confirm that perinatal malnutrition induced via this route has enduring consequences for brain and behavioural development^4^. Furthermore, recent studies show a role for perinatal malnutrition in the genesis of behavioural despair as measured by forced swim and tail suspension tests, suggesting that early-life nutrition could be important in the development of depression-like phenotypes^13,14^.

Food is arguably the most important developmental input. In addition to the fundamental role of food in fuelling normal growth and development, food is also a primary reinforcer, capable of shaping behaviour via learning. In mammals and altricial birds, parents provide their offspring with food until they are able to forage for themselves and thus control the feeding schedule experienced by their young in early life. In addition to the overall amount of food provided, parental feeding schedules may also differ in other respects such as the percentage of requests for food that are satisfied (i.e. the probability of reinforcement). Thus feeding schedules influence experience of reward, but also experience of punishment when an expected feed is omitted or delayed (an example of so-called negative punishment, or punishment by removal). We therefore hypothesised that early-life feeding schedules could be centrally important in calibrating the way individuals seek, evaluate and respond to reward and punishment. Furthermore, since depression is characterised by both hypo-responsivity to rewarding stimuli^15–17^ and hyper-responsivity to negative or punishing stimuli^18^, we hypothesised that early-life feeding schedules could be important in the development of depression-like phenotypes. The goal of our research was therefore to ask whether manipulating early-life feeding schedules would produce changes in adult reward sensitivity characteristic of depression. In order to ask whether there were effects of feeding schedules in addition to simple nutrition, we specifically set out to dissociate effects of the overall amount of food delivered from the effort required to obtain it, and hence attempt to test further a specific effects model of the development of depression and depression-like phenotypes.

**Figure 2:**
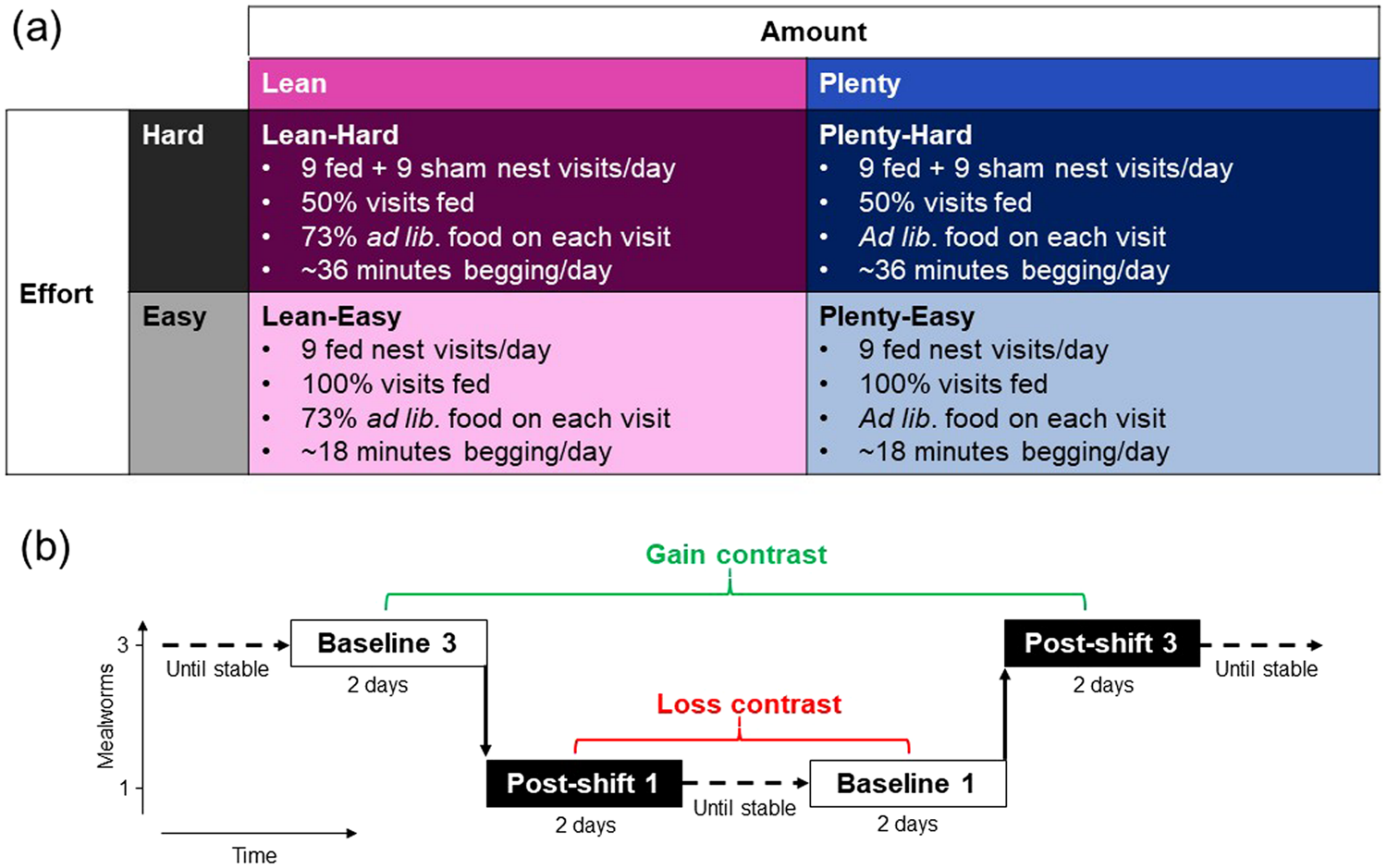
Experimental design. (**a**) Summary of the developmental manipulations reported fully in Nettle *et al*.^20^. (**b**) Summary of the successive positive/negative contrast experiment. All birds received the same sequence of experimental phases: Baseline 3, Post-shift 1, Baseline 1, Post-shift 3, where the number corresponds to the number of mealworms comprising the reward given in each trial of the experiment. Birds received 5 trials per day throughout, and were trained until their latencies to remove the lids were stable prior to Baselines 1 and 3 and following Post-shift 3. The data analysed are the latencies from the 8 days (=40 trials/bird) corresponding to the boxes. The Gain contrast (indicated in green) compared latencies in Baseline 3 and Post-shift 3, because in Post-shift 3 the birds have experienced a gain in reward (1 to 3), whereas the Loss contrast (indicated in red) compared latencies in Baseline 1 and Post-shift 1 because in Post-shift 1 the birds have experienced a loss in reward (3 to 1).

Dissociating different aspects of the early-life feeding schedule is inherently hard in young rodents that are difficult to hand-rear. However, hand-rearing is feasible in altricial bird species, such as the European starling (*Sturnus vulgaris*), because young chicks will readily imprint on a human caregiver^19^. We experimentally manipulated both the amount of food delivered to nestling starlings (Amount treatment) and the begging effort required to obtain the food (Effort treatment), in a factorial design implemented between days 6 and 15 post-hatching^20^ (see Fig. 2a). The Amount treatment comprised two levels of food amount: *ad libitum* food (henceforth Plenty) versus restriction to 73% of the average *ad libitum* amount (Lean). The Effort treatment comprised two levels of begging effort: birds fed on every ‘nest’ visit (Easy) versus birds fed on 50% of ‘nest’ visits (Hard). Thus, there were four treatment combinations in all: Lean Hard, Lean Easy, Plenty Hard and Plenty Easy.

We assessed sensitivity to reward in the adult starlings (9-13 months old at the start of the current experiment) using a combined, instrumental, successive negative contrast (SNC) and successive positive contrast (SPC) task^21^ (see Fig. 2b). In our version of the SNC/SPC task birds were required to remove a weighted lid to access a reward of either one or three mealworms. The latency to remove the lid provided a measure of motivation to access a given reward (i.e. its incentive value) and allowed us to assess how this motivation responded to sudden changes in reward value (loss or gain). A negative contrast effect is a disappointment-like response typically observed following a shift from a larger to a smaller reward, whereby motivation decreases, temporarily falling below the level seen in individuals exposed to the smaller reward for a prolonged period (the relevant comparison is indicated by the loss contrast in Fig. 2b). Conversely, a positive contrast effect is an elation-like response typically observed following a shift from a smaller to a larger reward, whereby motivation increases, temporarily rising above the level seen in individuals that have been exposed to the larger reward for a prolonged period^21,22^ (gain contrast in Fig. 2b). Contrast effects are proposed to be mediated by an emotional response to the shift in reward magnitude, and furthermore, there is evidence that the size and/or duration of these effects are moderated by the longer-term affective state of the individual, with larger and/or longer negative contrast effects and smaller and/or shorter positive contrast effects being observed in individuals in more negative affective states^23^. For example, rats that had been kept in unenriched cages showed a more prolonged negative contrast effect following reward loss compared with rats that had been kept in enriched cages^24^. The influence of affective state on contrast effects is additionally supported by evidence from pharmacological studies in rats showing that anxiolytic drugs such as benzodiazepines, which are assumed to reduce negative affect, reduce the magnitude of negative contrast effects^25^, whereas withdrawal from amphetamines, which is assumed to reduce positive affect, reduces positive contrast effects compared with controls^26^.

On the basis of the literature reviewed above we predicted that overall, birds should show a disappointment-like response to reward loss, and an elation-like response to reward gain, and furthermore, that the size of these contrast effects would be moderated by the developmental treatment to which a bird had been exposed. We predicted that birds that had experienced greater early-life adversity (i.e. Lean and/or Hard birds) would be most sensitive to reward loss and least sensitive to reward gain, whereas birds that had experienced less early-life adversity (i.e. Plenty and/or Easy birds) would be least sensitive to reward loss and most sensitive to reward gain. Under the common pathway model, both sources of adversity would be predicted to have similar effects on disappointment-like and elation-like responses, whereas under a specific effects model, differing effects of food deprivation and begging effort on disappointment-like and elation-like responses would be predicted. We were agnostic over whether we would see non-additive interactions between the two sources of adversity. Under the simple version of the specific effects model depicted in Fig. 1b we would not expect interactions; effects of different sources of adversity should be additive.

## Results

### Weight and body condition

There was no significant interaction between the developmental Effort and Amount treatments on adult body weight (and the interaction term was thus removed from the final model). However, the Effort treatment (experienced by the birds between days 6–15 post-hatching), still affected body weight at the start of the current experiment, with Hard birds lighter than Easy birds (Hard: mean ± se = 79.8 ± 1.50 g, n = 14; Easy: mean ± se = 86.96 ± 1.70 g, n = 16; Table 1, Model 1; Effort effect: LRT = 6.39, p = 0.0115). The Amount treatment had no effect on current body weight (Table 1, model 1). These effects remained after controlling for skeletal size (estimated by tarsus length), with Hard birds relatively light for their skeletal size (Hard: mean ± se = −3.74 ± 1.39 g, n = 14; Easy: mean ± se = 3.27 ± 1.62 g, n = 16; Table 1, Model 2; Effort effect: LRT = 4.79, p = 0.0286).

**Table 1:** Linear mixed model results.

Model	Response variable	Data set	No. birds	Predictor variables fixed	β	SE	LRT	P-value
1	Body weight at start (g)	All	30	Sex: male^1^	2.37	2.71	0.84	0.3599
				Effort: Easy^2^	6.41	2.16	6.39	0.0115*
				Amount: Plenty^3^	2.98	2.41	2.10	0.1475
2	Body weight at start (g)	All	30	Tarsus length	3.28	1.50	4.79	0.0286*
				Sex: male	−0.24	2.66	0.01	0.9218
				Effort: Easy	7.43	2.19	9.53	0.0020*
				Amount: Plenty	−1.01	2.63	0.17	0.6823
3	Log (Latency + 1) (s)	All	30	Effort: Easy	0.35	0.16	4.24	0.0394*
				Amount: Plenty	0.15	0.16	0.83	0.3614
4	Log (Latency + 1) (s)	All	30	Position: Post-shift^4^	0.06	0.03		
				Contrast: Gain^5^	0.12	0.03		
				Position × Contrast	−0.07	0.04	3.56	0.0592
4.1	Log (Latency + 1) (s)	Loss	30	Position: Post-shift	0.06	0.02	5.25	0.0220*
4.2	Log (Latency + 1) (s)	Gain	30	Position: Post-shift	−0.02	0.03	0.32	0.5743
4.3	Log (Latency + 1) (s)	All	30	Position: Post-shift	0.02	0.02	1.07	0.3006
				Contrast: Gain	0.09	0.02	20.67	< 0.0001*
5	Log (Latency + 1) (s)	All	30	Effort: Easy	0.33	0.24		
				Amount: Plenty	0.11	0.25		
				Position: Post-shift	0.04	0.05		
				Contrast: Gain	0.15	0.05		
				Effort × Amount	0.13	0.33		
				Effort × Position	−0.06	0.07		
				Effort × Contrast	−0.06	0.05		
				Amount × Position	0.13	0.07		
				Amount × Contrast	0.01	0.05		
				Position × Contrast	−0.06	0.07		
				Effort × Position × Contrast	0.15	0.08	4.05	0.0442*
				Amount × Position × Contrast	−0.17	0.08	4.83	0.0280 *
				Position × Effort × Amount	−0.23	0.08	8.946	0.0028*
5.1	Log (Latency + 1) (s)	Hard-Loss	14	Position: Post-shift^4^	0.12	0.03	5.59	< 0.0001*
5.2	Log (Latency + 1) (s)	Hard-Gain	14	Position: Post-shift^4^	−0.04	0.04	1.15	0.2828
5.3	Log (Latency + 1) (s)	Easy-Loss	16	Position: Post-shift^4^	0.00	0.04	0.01	0.9348
5.4	Log (Latency + 1) (s)	Easy-Gain	16	Position: Post-shift^4^	0.01	0.04	0.04	0.8492
5.5	Log (Latency + 1) (s)	Lean-Loss	14	Position: Post-shift^4^	0.05	0.03	2.18	0.1400
5.6	Log (Latency + 1) (s)	Lean-Gain	14	Position: Post-shift^4^	0.07	0.04	2.87	0.0902
5.7	Log (Latency + 1) (s)	Plenty-Loss	16	Position: Post-shift^4^	0.06	0.04	3.08	0.0794
5.8	Log (Latency + 1) (s)	Plenty-Gain	16	Position: Post-shift^4^	−0.09	0.04	5.31	0.0213*

Notes: The models presented in this table are simplified models from which non-significant interaction terms have been removed. All models contain a random effect of natal family and models 3–5.8 additionally contain a random effect of individual bird. ^1^reference group for Male is Female; ^2^reference group for Easy is Hard; ^3^reference group for Plenty is Lean; ^4^reference group for Post-shift is Baseline; ^5^reference group for Gain is Loss. P-values are from the likelihood ratio test; *p < 0.05.

### Latencies to remove lids

#### Overall effect of developmental treatment

**Figure 3:**
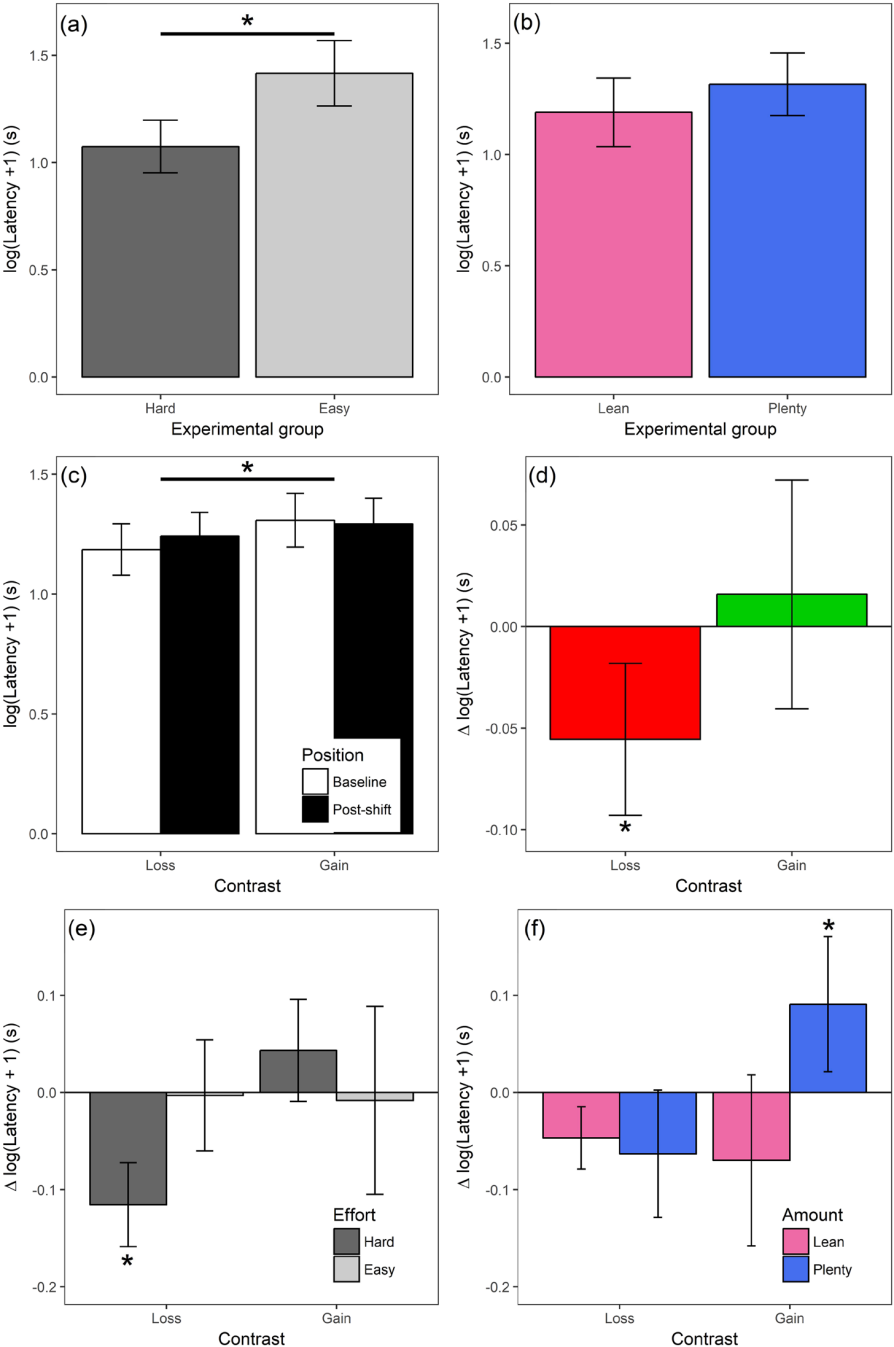
Behavioural data: latencies to remove lids. Bars represent means and error bars one standard error (based on the number of birds in each group). All panels are based on data from 30 birds. Asterisks (*) indicate significant differences (p < 0.05); in panels a and c the significant differences are between groups (as indicated by the bars), whereas in panels d, e and f the marked bar is significantly different from zero. Note that for clarity, the colours used to designate the different groups in this figure map onto the colours used to represent the different treatments, phases and comparison in Fig. 2. (**a**) Latencies for the pooled data from all four phases of the experiment (Baseline 3, Post-shift 1, Baseline 1 and Post-shift 3) split by the two levels of the Effort developmental treatment. (**b**) Latencies for the pooled data from all four phases of the experiment split by the two levels of the Amount developmental treatment. (**c**) Latencies for the pooled data from all birds split by the four phases of the experiment. (**d**) The same data shown in panel (**c**) expressed as Δ latency calculated by subtracting the mean post-shift latency for each bird from its mean baseline latency; negative values represent slowing post-shift, whereas positive values represent speeding up. (**e**) Δ latency split by Contrast type and levels of the Effort treatment. (**f**) Δ latency split by Contrast type and levels of the Amount treatment. Note that in panels a, b, e and f the data are split by the main effect only, since no significant interactions were found between Effort and Amount.

To explore the effects of developmental treatment on motivation to access mealworms we modelled the effects of Effort, Amount and the Effort x Amount interaction on latency pooled across all four phases of the experiment (Baseline 1 and 3 and Post-shift 1 and 3; Fig. 2b). There was no significant interaction between Effort and Amount on latency to remove lids (and the interaction term was thus removed from the final model). However, the Effort treatment affected latency to remove lids, with Hard birds faster than Easy birds (Fig. 3a and Table 1, model 3; Effort effect: LRT = 4.24, 0.0394); the Amount treatment had no overall effect on latency (Fig. 3b and Table 1, model 3).

#### Overall effect of reward loss and gain

In our analyses, the predictor variable called Position refers to whether a latency is from a baseline or post-shift phase (white and black boxes respectively in Fig. 2b), and the predictor variable called Contrast refers to whether a latency is from the loss or gain comparison (indicated in red and green respectively in Fig. 2b). Thus, the typical finding of a negative contrast effect with reward loss and a positive contrast effect with reward gain would be reflected in a significant Position x Contrast interaction effect on latencies. To explore whether our novel instrumental SNC/SPC task produced negative and/or positive contrast effects, we modelled the effects of Position (i.e. Baseline versus Post-shift) and Contrast (i.e. Loss versus Gain) on latencies pooled across all 30 birds. There was a marginally non-significant Position x Contrast interaction effect on latency (Fig. 3c and Table 1, model 4; Position x Contrast interaction effect: LRT = 3.56, p = 0.0592). To understand this interaction further, we split the data into two subsets corresponding to the Loss and Gain contrasts. In each of these subsets we analysed the effect of Position on latency to determine whether birds slowed down or sped up following a loss or gain. These post-hoc analyses revealed an effect of Position in the Loss condition with latencies slowing following a loss in reward (Fig. 3d and Table 1, model 4.1; LRT = 5.25, p = 0.0220); however, there was no effect of Position in the Gain condition (Fig. 3d Table 1, model 4.2). Removing the marginally non-significant Position x Contrast interaction from model 4 revealed a main effect of Contrast, with faster latencies overall in the Loss condition (in which the reward was 1 mealworm) than the Gain condition (where the reward was 3 mealworms; Fig. 3c and Table 1, model 4.3; Contrast effect: LRT = 20.67, p < 0.0001); there was no main effect of Position.

#### Interaction between developmental treatment and contrast

Finally, to explore whether developmental treatment affected how birds responded to loss and gain, we entered developmental treatment (Effort, Amount), Position, Contrast and their interactions as predictor variables in the same model of latency. The 4-way interaction (Position x Contrast x Effort x Amount) and the 3-way interaction between Contrast x Effort x Amount were non-significant and were removed from the final model. There were three 3-way interaction effects on latency: Position x Effort x Amount, Position x Contrast x Effort and Position x Contrast x Amount (Table 1, model 5; Position x Effort x amount interaction: LRT = 8.95, p = 0.0028; Position x Contrast x Effort interaction: LRT = 4.05, p = 0.0442; Position x Contrast x Amount interaction: LRT = 4.83, p = 0.0280). To interpret the interactions relating to effects of developmental treatment on response to reward loss and gain, we split the data into four subsets for the Effort treatment (Hard-Loss, Hard-Gain, Easy-Loss and Easy-Gain) and four subsets for the Amount treatment (Lean-Loss, Lean-Gain, Plenty-Loss and Plenty-Gain), and in each of these subsets analysed the effect of Position on latency to determine whether birds from individual treatments slowed down or sped up when the reward value shifted. These post-hoc analyses yielded two models with effects of Position: Hard birds slowed down between Baseline and Post-shift when the post-shift reward represented a Loss (Fig. 3d and Table 1, model 5.1; Position effect: LRT = 16.24, p < 0.0001), and Plenty birds sped up between Baseline and Post-shift when the post-shift reward represented a Gain (Fig. 3e and Table 1, model 5.8; Position effect: LRT = 5.31, p = 0.0213); none of the other six models revealed an effect of Position (Fig. 3d,e and Table 1, models 5.2–5.7).

## Discussion

We investigated the effect of two types of early-life adversity, namely food deprivation (Lean Amount) and high begging effort (Hard Effort), on sensitivity to reward loss and gain in adult starlings. Developmental begging effort affected birds’ overall motivation to obtain reward, with birds that had experienced high begging effort as nestlings (Hard group) being more motivated than birds that had experienced low begging effort (Easy group) to obtain a food reward in all phases of the experiment. Both the Effort and Amount treatments affected sensitivity to change in the value of reward, with only Hard birds showing a decline in motivation following a loss in reward, and only Plenty birds showing an increase in motivation following a gain in reward. Thus, the Hard birds demonstrated a negative contrast effect following loss, sometimes referred to as ‘disappointment’, that was not seen in the Easy birds, whereas the Plenty birds demonstrated a positive contrast effect following gain, sometimes referred to as ‘elation’, that was not seen in the Lean birds. These results demonstrate that the feeding schedule experienced for just 10 days in early life can cause enduring effects on adult feeding motivation and sensitivity to reward loss and gain. Furthermore, these effects appear to be specific to different types of early-life adversity, with high begging effort being implicated in increased adult feeding motivation and a higher propensity to exhibit ‘disappointment’ following a loss, but food deprivation being implicated in a lower propensity to exhibit ‘elation’ (a kind of anhedonia) following a gain. There was no evidence for interactions between Effort and Amount in their effects on adult behaviour; the effects were additive. Our results therefore support a specific effects model of the development of depression-like traits as depicted in Fig. 1b.

Before going on to discuss the interpretation of the above findings, we first address some methodological issues relating to our behavioural task. The task we employed differed from existing SNC/SPC tasks in two respects. First, we assessed contrast effects within subjects rather than comparing separate baseline and shifted groups^22,24^. This within-subjects approach allowed us to measure contrast effects in all of our developmentally manipulated birds and to control better for between-subject differences in motivation that would add noise in a traditional design; the disadvantage was that extensive training was necessary to obtain stable baseline latencies prior to the baseline measurements, making the method time-consuming. Second, in contrast to previous studies we used latency to remove a weighted lid as our behavioural measure of motivation. We assumed that shorter latencies reflected higher motivation. In contrast to previous studies, where motivation typically increases with incentive value, latencies in our task were absolutely faster overall in the loss phases of the experiment with a one-mealworm reward compared to the gain phases with a three-mealworm reward. This result most likely reflects the fact that the birds were more acutely food deprived, and thus more motivated to obtain food, when the size of the reward was smaller. In an attempt to equalise food deprivation levels across phases, we followed the usual protocol adopted in this literature of equalising the number of rewards delivered to each individual each day by giving 10 supplementary mealworms an hour after the end of each session of one-mealworm trials^22,24^. However, we suspect that this post-session supplementation was not sufficient to equalise deprivation in the one- and three-mealworm phases due to the number of trials we ran per session (five) and the length of the inter-trial interval (21 minutes) resulting in a total session duration of around 2 hours (note that Crespi^22^ avoided the problem of deprivation building up over the session by running only a single trial each day, which was not logistically possible in our study due to the extensive training needed to achieve baseline stability). Although it is therefore likely that the birds were more acutely food deprived in the loss condition than the gain condition, the effects of developmental treatment on contrast effects relate to the results obtained within each of these conditions and are thus not confounded by between-condition differences in motivation.

Overall, the birds slowed relative to baseline performance following a reward loss, but showed no change in latency relative to baseline performance following a reward gain. Thus, we obtained a negative contrast effect in response to reward loss, but no significant positive contrast effect in response to reward gain. These findings are typical of those from other species and SNC/SPC tasks: negative contrast effects in response to loss are a ubiquitous finding in the literature, whereas positive contrast effects in response to gain are far less robust and often appear to depend on details of the methodology^21^. Based on a review of the literature, Flaherty^21^ has argued that greater acute food deprivation may favour the occurrence of positive contrast effects. Thus, if our birds were less deprived in the gain comparison than the loss comparison, as we have argued above is likely, this could account for why we did not observe a significant overall positive contrast effect in the gain comparison with our task. In summary, it is reassuring that despite the methodological differences between our task and previous SNC/SPC tasks discussed above, our overall results are broadly comparable.

In the following paragraphs we discuss the effects of the developmental manipulations of adversity on the adult phenotypes of the birds. The effect of the high begging effort developmental treatment was still evident in the body weights of the birds at the start of the current experiment. Hard birds were both absolutely lighter than Easy birds, and also lighter for their skeletal size at 9–12 months of age, despite having been maintained on identical feeding regimes since the end of the developmental manipulation on day 15. Interestingly, the effect of developmental food deprivation on body weight seen at day 56 and reported previously^20^ had disappeared in the current study: there was no enduring weight difference between Lean and Plenty birds. In a previous cohort of starlings in which we manipulated early-life adversity by cross-fostering chicks into nests where they were either the smallest or largest chicks^19^, we also found a long-term effect of the manipulation on adult body condition. However, the result was in the opposite direction to the effect of high begging effort reported here: birds disadvantaged as chicks, by being smaller than their nest mates, had higher body condition (i.e. they were relatively heavier for their skeletal size) as adults^27^. We currently have no explanation for this difference. However, it is worth pointing out that a review of the rodent literature on effects of early-life adversity on adult body weight also found no consistent direction to the effects reported^11^. Furthermore, the DSM-IV^28^ criteria for major depressive disorder in humans include, “Significant weight change (5%) or change in appetite”, with no reference to the direction of this change. Thus, either reduced or increased body weight and appetite are characteristic of early-life adversity in animal models and of depression in humans. This inconsistency in effects on body weight suggests that differences in the specific nature of adverse experience not thus far identified are likely to be important in determining the direction of the effects.

The results from the SNC/SPC task established that the enduring effects of early-life adversity in our starlings are not restricted to body weight, but can also be seen in various aspects of their foraging motivation. Hard birds were overall more motivated to obtain food than Easy birds, behaving as if they were acutely food deprived compared with the Easy birds, despite having been reared in identical conditions since the end of the manipulation. As discussed above, increased appetite is a criterion for human depression, meaning that this effect is compatible with a depression-like phenotype in the Hard birds. Similar effects of early-life experience on adult food motivation have previously been observed in European starlings. Birds that experienced greater competition for food as chicks displayed hyperphagy when food deprived, invested more effort in acquiring information about the location of food, and were more prepared to consume unpalatable prey^27,29^. It is interesting to speculate why Hard birds specifically should behave as if they are acutely food deprived. There is a literature showing that animals value a specific reward more highly if they have had to work harder to obtain it in the immediate past^30,31^. Our research raises the possibility that the effort expended acquiring food in early life could have a lasting impact on the value adult animals assign to food and hence their feeding motivation.

Few previous studies have explored the effects of manipulations of early-life adversity on subsequent behaviour on SNC/SPC tasks. No effects of early human handling were found on either instrumental or consummatory SNC effects in rats^32,33^. Flaherty^21^ speculated that this might be because this manipulation produced differences in adult fearfulness, as opposed to reward sensitivity as measured by the SNC/SPC task. More recently, a single study showed that maternal separation produced a blunted consummatory SPC effect in rats, suggestive of an anhedonic, depression-like phenotype similar to that seen in our Lean birds^34^. However, the same animals in the latter study also showed blunted SNC effects, which is the opposite of what is seen in human depression. Our study is therefore unique in that for the first time in any developmental animal model of depression we produced adult animals (specifically the Lean-Hard treatment combination) with the depression-like signature of both an enhanced disappointment-like response to reward loss (SNC) and a reduced elation-like response to reward gain (SPC). High begging effort (in the Hard birds) enhanced the SNC effect, whereas food deprivation (in the Lean birds) abolished the SPC effect. High begging effort and food deprivation thus had additive effects, but there were no non-additive interactions, supporting a specific effects model of the effects of developmental adversity on contrast effects.

Why, mechanistically, should higher begging effort and food deprivation have different effects on SNC and SPC? One possibility is that high begging effort and food deprivation specifically affected the respective development of the birds’ negative and positive affect systems. We propose that the Hard begging effort treatment exposed developing chicks to repeated negative punishment via non-delivery of expected food, and that this history of punishment produced adult birds with greater negative affect characteristic of a more anxious phenotype^35^. In contrast the Lean food amount treatment exposed developing chicks to a chronic lack of rewards, and this history produced adult birds with reduced positive affect characteristic of a more depression-like phenotype^36,37^. Corroborating evidence for a more anxious phenotype in the Hard birds indicative of greater negative affect comes from data showing that these birds spontaneously adopted higher (and hence presumably safer) perching positions in the aviary than their Easy siblings^38^.

We have described the phenotypic outcomes of enhanced ‘disappointment’ and reduced ‘elation’ as depression-like, on the basis of their similarity to characteristics seen in clinical depression in humans. We do not mean to imply thereby that they can only be interpreted as pathological. When specific phenotypic characteristics are reliably caused by early experience, pathology due to developmental insult is only one possible explanation. Alternatives are that the characteristics represent a beneficial phenotype for the environment the individual is statistically likely to encounter as an adult (the external predictive adaptive response hypothesis)^39^; or that the characteristics are beneficial given the somatic states—such as size, strength or dominance—the individual is likely to have as an adult (the internal predictive adaptive response hypothesis)^19^. Several authors have argued that depression-like cognitive attributes may be beneficial, conditional on particular states of the environment or of the individual’s capacities^36,40–42^. Thus, it is possible that the enduring changes in responsiveness to reward and punishment as a consequence of particular early experiences documented here represent adaptive responses. This possibility is reinforced by the fact that our experimental treatments produced experiences that fell within the recurrent natural range for wild starlings. However, our current results do not establish that the phenotypes are adaptive, still less why. To do so would require further research into the naturalistic consequences of different levels of responsiveness to changes in reward and punishment under varying adult circumstances.

In conclusion, our results support the hypothesis that early-life feeding schedules are important in the development of depression-like phenotypes, but show that the specifics of what is experienced can be critically important in determining the developmental outcome. In epidemiological studies of the correlates of early life adversity, different types of adversity (e.g. physical and emotional neglect) are often summed to yield a single adversity score^43–45^. Such methods may mask unique causal signatures of different types of adversity^6^. Anxiety and depression have high comorbidity in humans^46^, which has been taken to suggest a common mechanism^47–49^. However, our results suggest an alternative possibility, which is that both disorders could have different causes (relating respectively to experience of punishment and experience of reward), but that these causes tend to co-occur in un-manipulated human environments. More generally, our results contribute to recent arguments that the role played by feeding schedules in shaping adult human behaviour may have been underestimated and needs serious consideration^50,51^.

## Methods

### Subjects

Subjects were 31 hand-reared European starlings removed from nests in the wild on day 5 post-hatching (15 females and 16 males established via molecular sexing). This research was conducted under licence from the UK Home Office (PPL 60/4073) and removal of European starlings from the wild was authorised by Natural England (licence number 20121066). The study adhered to ASAB/ABS guidelines for the use of animals in research.

The birds originally comprised eight families of four siblings approximately matched for weight on day 5. As nestlings, the birds were subjected to an experimental manipulation of feeding during the hand-rearing period, the details of which have been previously described^20^. In brief, the manipulation followed a two-by-two factorial design in which both Amount of food and the begging Effort required to obtain it were varied between individuals for 10 days between day 6 and day 15 post-hatch (Fig. 2a). One bird from each family of siblings was assigned to each treatment combination. In the food Amount treatment, half the nestlings were fed to satiation every feed (Plenty). The other half (Lean) were given a fixed ration equal to a proportion of the mean amount consumed by the Plenty groups at that feed. This proportion was initially set to 70%, but dynamically adjusted each day so that the weight gain of the Lean birds tracked the lightest nestlings in an earlier study of wild-reared nestlings^52^, ending up at an average of 73% over the course of the whole manipulation. In the begging Effort treatment, half the nestlings were fed on every feed (Easy) and the remaining half received twice the number of nest visits but were only fed on half of these, being stimulated to beg for an equivalent time period on alternate non-fed visits (Hard). There were thus four treatment groups of eight birds: Plenty Easy; Plenty Hard; Lean Easy and Lean Hard. After day 15 the birds were all fed to satiation on every feed until fledging. Following fledging (around day 21), the birds were group-housed in an indoor aviary (215 × 340 × 220 cm WDH; ~18 °C; 40% humidity; 15 L: 9D light cycle) and provided with *ad libitum* food and water. The adult diet, fed from fledging onwards, comprised cat biscuit, domestic chick crumb, and fruit. The 15-hour days in the laboratory insured that the birds were maintained in non-reproductive condition, with regressed gonads, throughout the period of study. Note that there were initially 32 birds but one bird (Lean Hard) died in adulthood in an aviary accident prior to the current experiment. The distribution of sexes among treatments was as follows: Lean: 4 females, 11 males; Plenty: 11 females, 5 males; Hard: 11 females, 4 males; Easy: 4 females, 12 males).

The birds were 9–13 months old at the start of the current experiment. They completed the experiment in four successive replicates each of which comprised two families (i.e. seven or eight birds). Each replicate was caught and moved from the aviary to individual home cages (100 × 45 × 45 cm WDH) in an adjacent experimental room (~18 °C; 40% humidity; 15 L: 9D light cycle) two days prior to the start of the experiment. Whilst in individual cages, the birds remained in auditory contact despite being visually separated by dividers between each cage. Birds were provided with *ad libitum* access to food (adult diet as described above) for 20.5 hours each day. Drinking water was available *ad libitum*. Environmental enrichment was provided in the form of two perches and a bath. The bath was removed for the duration of the daily sessions. Each replicate remained in individual cages for no more than four weeks and was returned to the indoor aviary following completion of the experiment.

### Behavioural Task

For the behavioural task, birds were required to progress through lid flipping training followed by four experimental phases as shown in Fig. 2b. In all phases, food was removed from the home cages immediately before the lights came on in the morning. Birds began the task 30 minutes later.

During lid-flipping training, subjects were trained to remove a lid from the top of a Petri dish to obtain a food reward. The lid-flipping task and training regime used a modified version of the methodology developed by Bateson and colleagues^53,54^. Throughout training, subjects were presented with an opaque Petri dish containing three mealworms, attached to a white ceramic tile, placed in the centre of the cage. A yellow, circular cardboard lid was also placed in the cage, with its position varying depending on the stage of training. Initially the lid was placed beside the Petri dish, then leant against the Petri dish, then moved to partially cover the Petri dish, before finally the Petri dish was completely covered by the lid. Following this stage, lids were weighted with between one and three metal washers attached to the underside in order to progressively increase the effort required to remove the lid. At the start of training, lids were weighted with 33% of the final weight used, which was increased to 66%, and finally 100% after successful removal of the lid completely covering the Petri dish. The final total weight of the lid was approximately 43 g, a weight that had been determined through a pilot study to require time and effort to remove, but for removal to still be possible for every bird. To progress through each stage, subjects were required to consume all mealworms presented. If unsuccessful, subjects would repeat their current stage. Subjects were given a maximum of 15 minutes to consume the mealworms, and were given five trials per day with an inter-trial interval (ITI) of two minutes. Subjects in the same replicate were presented with the Petri dishes and lids near-simultaneously. Subjects progressed to the experimental phases once all individuals in the replicate had successfully removed the fully-weighted lid from the Petri dish.

In all subsequent sessions, subjects in the same replicate were presented with the Petri dish and lid consecutively, with a trial duration of three minutes, an ITI of 21 minutes, and five trials per day. In the phase immediately following training, Baseline 3, the fully-weighted lid was placed on top of the Petri dish containing three mealworms. Subjects progressed onto the next phase, the SNC task, when their latency to remove the lid had stabilised. A stable latency was defined as two consecutive sessions in which the average latency across the five trials did not change by more than 10%, or in which the absolute change was less than 0.05 seconds.

Once latency to obtain the reward had stabilised, the number of mealworms given for removing the lid was reduced to one (Post-shift 1). Following the methodology of Burman *et al*.^24^, subjects in the Post-shift 1 and Baseline 1 phases received a supplementary 10 mealworms at least an hour after the end of their final trial to ensure that the total amount of food received per day throughout the experiment remained constant. Subjects progressed to the Baseline 1 phase once the latency to remove the lid from the Petri dish had stabilised. In the final phase, the number of mealworms rewarded for removing the lid was increased to three (Post-shift 3). Subjects finished the experiment once they reached the stability criterion following reward gain.

### Data Collection

Sessions were recorded using video cameras facing the home cages. The latency of each subject to remove the lid from the Petri dish was scored using JWatcher (0.9) software^55^. The latency was defined as the time period starting when the cage door closed following the experimenter placing the Petri dish in the cage, and ending when the bird removed the lid sufficiently to access the mealworm(s). One session from each replicate was re-scored to check intra-observer reliability. Subjects were required to consume at least 80% of the mealworms in the two sessions prior to and following the shift.

### Data Analysis

The response variables analysed were: the adult body weights of the birds at the start of the current experiment and the latencies of the birds to remove lids in the positive/negative contrast task. One bird (Lean Hard male) did not meet the mealworm consumption criterion in the contrast task and was excluded from all the analyses; this did not qualitatively affect the results.

We analysed these data using general linear mixed models (GLMM) implemented in the R package ‘nlme’. Models were fitted using maximum likelihood estimation. We used a likelihood-ratio test (LRT), which tests the difference in model deviance (χ^2^ distributed) when a predictor variable is removed from the model, to determine whether parameter estimates differed significantly from zero^56^. In all models in which latency to remove the lid was the response variable, a value of 1 was added to the trial latency and then log transformed to ensure that the model assumptions of normality and homogeneity of variance were not violated. The residuals of each model were visually inspected to verify that the transformation was appropriate. The criterion for significance was p < 0.05, and results with p < 0.075 were considered as marginally non-significant trends.

To deal with non-independence due to siblings coming from the same family and due to repeated measures of the same individual, all models included random effects of family and individual (where appropriate), with individual nested in family. To reflect the factorial nature of our experimental design, all models initially included interaction terms: Effort x Amount (models 1, 2, 3 and 5), Position x Contrast (model 4) and Effort x Amount x Position x Contrast (model 5; see accompanying R script). However, if the highest order interaction in a model was not significant, then this term was removed from the final model presented in Table 1 in order to maximise the power available to test main effects. Since sex was not balanced across treatment groups, to test whether sex could explain our findings, we explored the effects of sex on latencies. We found no overall effect of sex on latency (model S0, Table S1). Moreover, adding sex as an additional predictor in models 3–5 made no qualitative difference to results obtained (models S3-S5, Table S1). Therefore, sex was not included in the models presented in Table 1.

### Data availability

All data generated or analysed during this study and an R script that replicates all of the analyses reported in this article are freely available via the Zenodo repository: https://doi.org/10.5281/zenodo.844837.

## Electronic supplementary material

Table S1
