# Supplementary material for: Dissociating the effects of alternative early-life feeding schedules on the development of adult depression-like phenotypes
Source: Sci Rep. 2017 Nov 1;7:14832. doi: 10.1038/s41598-017-13776-4 (PMC5665890; doi:10.1038/s41598-017-13776-4)
Supplement: Supplementary file 1 — Table S1 [file 41598_2017_13776_MOESM1_ESM.doc]

**Dissociating the effects of alternative early-life feeding schedules on the development of adult depression-like phenotypes.**

Vikki Neville^1, 2^, Clare Andrews^1^, Daniel Nettle^1^ and Melissa Bateson^1,*^

^1^Institute of Neuroscience and Centre for Behaviour and Evolution, Newcastle University, Henry Wellcome Building, Framlington Place, Newcastle upon Tyne, NE2 4HH.

^2^Current affiliation: School of Veterinary Science, University of Bristol, UK.

^*^Corresponding author. Email: [Melissa.Bateson@ncl.ac.uk](mailto:Melissa.Bateson@ncl.ac.uk)

**Supplementary information: Table S1**

**Table S1. Linear mixed model results controlling for sex**

| **Model** | **Response variable** | **Data set** | **No. birds** | **Predictor variables fixed** | **β** | **SE** | **LRT** | **P-value** |
| --- | --- | --- | --- | --- | --- | --- | --- | --- |
| S0 | Log (Latency +1) (s) | All | 30 | Sex: Male | -0.15 | 0.18 | 0.65 | 0.4204 |
| S3 | Log (Latency +1) (s) | All | 30 | Effort: Easy | 0.62 | 0.18 | 9.52 | 0.0020 * |
|  |  |  |  | Amount: Plenty | -0.03 | 0.17 | 0.03 | 0.8545 |
|  |  |  |  | Sex: Male | -0.51 | 0.21 | 5.61 | 0.0178 * |
| S4 | Log (Latency +1) (s) | All | 30 | Position: Post-shift | 0.06 | 0.03 |  |  |
|  |  |  |  | Contrast: Gain^5^ | 0.12 | 0.03 |  |  |
|  |  |  |  | Position x Contrast | -0.07 | 0.04 | 3.56 | 0.0593 |
|  |  |  |  | Sex: Male | -0.15 | 0.18 | 0.65 | 0.4206 |
| S4.1 | Log (Latency +1) (s) | Loss | 30 | Position: Post-shift | 0.06 | 0.02 | 5.25 | 0.0220 * |
|  |  |  |  | Sex: Male | -0.15 | 0.18 | 0.68 | 0.4090 |
| S4.2 | Log (Latency +1) (s) | Gain | 30 | Position: Post-shift | -0.02 | 0.03 | 0.31 | 0.5747 |
|  |  |  |  | Sex: Male | -0.15 | 0.19 | 0.60 | 0.4380 |
| S4.3 | Log (Latency +1) (s) | All | 30 | Position: Post-shift | 0.02 | 0.02 | 1.07 | 0.3006 |
|  |  |  |  | Contrast: Gain | 0.09 | 0.02 | 20.67 | <0.0001 * |
|  |  |  |  | Sex: Male | -0.15 | 0.18 | 0.65 | 0.4203 |
| S5 | Log (Latency +1) (s) | All | 30 | Effort: Easy | 0.71 | 0.27 |  |  |
|  |  |  |  | Amount: Plenty | 0.10 | 0.25 |  |  |
|  |  |  |  | Position: Post-shift | 0.04 | 0.05 |  |  |
|  |  |  |  | Contrast: Gain | 0.15 | 0.05 |  |  |
|  |  |  |  | Effort x Amount | -0.05 | 0.31 |  |  |
|  |  |  |  | Effort x Position | 0.01 | 0.07 |  |  |
|  |  |  |  | Effort x Contrast | 0.01 | 0.07 |  |  |
|  |  |  |  | Amount x Position | 0.13 | 0.07 |  |  |
|  |  |  |  | Amount x Contrast | 0.01 | 0.05 |  |  |
|  |  |  |  | Position x Contrast | -0.06 | 0.07 |  |  |
|  |  |  |  | Effort x Position x Contrast | 0.15 | 0.08 | 4.05 | 0.0442 * |
|  |  |  |  | Amount x Position x Contrast | -0.17 | 0.08 | 4.83 | 0.0280 * |
|  |  |  |  | Position x Effort x Amount | -0.23 | 0.08 | 8.95 | 0.0028 * |
|  |  |  |  | Sex: Male | -0.54 | 0.21 | 5.89 | 0.0153 * |
| S5.1 | Log (Latency +1) (s) | Hard-Loss | 14 | Position: Post-shift^4^ | 0.12 | 0.03 | 16.24 | <0.0001 * |
|  |  |  |  | Sex: Male | -0.51 | 0.24 | 4.10 | 0.0428 * |
| S5.2 | Log (Latency +1) (s) | Hard-Gain | 14 | Position: Post-shift^4^ | -0.04 | 0.04 | 1.15 | 0.2832 |
|  |  |  |  | Sex: Male | -0.54 | 0.27 | 3.44 | 0.0635 |
| S5.3 | Log (Latency +1) (s) | Easy-Loss | 16 | Position: Post-shift^4^ | 0.00 | 0.04 | 0.01 | 0.9348 |
|  |  |  |  | Sex: Male | -0.52 | 0.31 | 2.44 | 0.1181 |
| S5.4 | Log (Latency +1) (s) | Easy-Gain | 16 | Position: Post-shift^4^ | 0.01 | 0.04 | 0.04 | 0.8482 |
|  |  |  |  | Sex: Male | -0.47 | 0.29 | 2.60 | 0.1070 |
| S5.5 | Log (Latency +1) (s) | Lean-Loss | 14 | Position: Post-shift^4^ | 0.05 | 0.03 | 2.18 | 0.1400 |
|  |  |  |  | Sex: Male | -0.02 | 0.31 | 0.00 | 0.9500 |
| S5.6 | Log (Latency +1) (s) | Lean-Gain | 14 | Position: Post-shift^4^ | 0.07 | 0.04 | 2.87 | 0.0902 |
|  |  |  |  | Sex: Male | 0.02 | 0.29 | 0.01 | 0.9367 |
| S5.7 | Log (Latency +1) (s) | Plenty-Loss | 16 | Position: Post-shift^4^ | 0.06 | 0.04 | 3.08 | 0.0794 |
|  |  |  |  | Sex: Male | -0.22 | 0.27 | 0.64 | 0.4247 |
| S5.8 | Log (Latency +1) (s) | Plenty-Gain | 16 | Position: Post-shift^4^ | -0.09 | 0.04 | 5.31 | 0.0213 * |
|  |  |  |  | Sex: Male | -0.33 | 0.31 | 1.10 | 0.2944 |

Notes: The models presented in this table are simplified models from which non-significant interaction terms have been removed. All models contain a random effect of natal family and models 3-5.8 additionally contain a random effect of individual bird. P-values are from the likelihood ratio test; *p < 0.05.
